# Distribution and Dispersal of *Phlebotomus papatasi* (Diptera: Psychodidae) in a Zoonotic Cutaneous Leishmaniasis Focus, the Northern Negev, Israel

**DOI:** 10.1371/journal.pntd.0004819

**Published:** 2016-07-18

**Authors:** Laor Orshan, Shirly Elbaz, Yossi Ben-Ari, Fouad Akad, Ohad Afik, Ira Ben-Avi, Debora Dias, Dan Ish-Shalom, Liora Studentsky, Irina Zonstein

**Affiliations:** 1 Laboratory of Entomology, Ministry of Health, Jerusalem, Israel; 2 Israel Nature and Parks Authority, Jerusalem, Israel; 3 Ministry of Environmental Protection, Southern District, Be'er Sheva, Israel; Institut Pasteur de Tunis, TUNISIA

## Abstract

**Background:**

Zoonotic cutaneous leishmaniasis has long been endemic in Israel. In recent years reported incidence of cutaneous leishmaniasis increased and endemic transmission is being observed in a growing number of communities in regions previously considered free of the disease. Here we report the results of an intensive sand fly study carried out in a new endemic focus of *Leishmania major*. The main objective was to establish a method and to generate a data set to determine the exposure risk, sand fly populations' dynamics and evaluate the efficacy of an attempt to create "cordon sanitaire" devoid of active jird burrows around the residential area.

**Methodology/Principal Findings:**

Sand flies were trapped in three fixed reference sites and an additional 52 varying sites. To mark sand flies in the field, sugar solutions containing different food dyes were sprayed on vegetation in five sites. The catch was counted, identified, *Leishmania* DNA was detected in pooled female samples and the presence of marked specimens was noted. *Phlebotomus papatasi*, the vector of *L*. *major* in the region was the sole *Phlebotomus* species in the catch. *Leishmania major* DNA was detected in ~10% of the pooled samples and the highest risk of transmission was in September. Only a few specimens were collected in the residential area while sand fly numbers often exceeded 1,000 per catch in the agricultural fields. The maximal travel distance recorded was 1.91km for females and 1.51km for males. The calculated mean distance traveled (MDT) was 0.75km.

**Conclusions:**

The overall results indicate the presence of dense and mobile sand fly populations in the study area. There seem to be numerous scattered sand fly microsites suitable for development and resting in the agricultural fields. Sand flies apparently moved in all directions, and reached the residential area from the surrounding agricultural fields. The travel distance noted in the current work, supported previous findings that *P*. *papatasi* like *P*. *ariasi*, can have a relatively long flight range and does not always stay near breeding sites. Following the results, the width of the "cordon sanitaire" in which actions against the reservoir rodents were planned, was extended into the depth of the agricultural fields.

## Author Summary

Cutaneous leishmaniasis is a dermal disease of public health concern in Israel. The typical skin lesions are caused by single celled *Leishmania* parasites that are transmitted by the bite of an infected female sand fly. In the last years, the disease has been spreading and emerged in Urim, a small cooperative community, surrounded by agricultural fields in southern Israel. In this area the transmission cycle of leishmaniasis involves rodents as reservoir animals. For the purpose of experimental intervention against the reservoir animals, it was necessary to find out from what areas and distances infected sand flies arrived to the residential areas. The research in 2013 was based on integrated methodical monitoring of sand flies using CO_2_ baited traps and on marking. The results indicated the existence of dense and mobile sand fly population around Urim. The agricultural fields seemed to be the major source for sand flies. Sand flies apparently moved in all directions to distances typically of at least 0.75km. The risk of exposure to *Leishmania*-infected sand fly females had a distinct seasonal peak in September. The information was used to determine the "cordon sanitaire" in which actions against the reservoir had to be carried out.

## Introduction

Zoonotic cutaneous leishmaniasis (ZCL) has long been endemic in Israel [1]. Two *Leishmania* species cause ZCL in Israel: *Leishmania major*, which is transmitted by *Phlebotomus (Phlebotomus) papatasi* (Scopoli), and *Leishmania tropica*, which is transmitted by *Phlebotomus (Paraphlebotomus) sergenti* Parrot and *Phlebotomus (Adlerius) arabicus* Theodor (Diptera: Psychodidae: Phlebotominae) [1–7]. The rodent reservoirs of *L*. *major* are sand rats (*Psammomys obesus* Cretzschmar), jirds (*Gerbillus dasyurus* Wagner, *Meriones crassus* Sundevall and *Meriones tristrami* (Thomas) and perhaps also voles [8–11], whereas rock hyraxes (*Procavia capensis* Pallas) are considered animal reservoirs of *L*. *tropica* in Israel [12].

The reported incidence rates of ZCL in Israel increased from 0.4/100,000 population in 2001 to 4.4/100,000 population in 2012 [13]. Endemic transmission has been observed in a growing number of communities in regions previously considered free of the disease [11, 13—14]. This situation and the lack of effective control methods led the Ministry of Environmental Protection and the Ministry of Health in 2012, to propose that the Israeli Government implement a pilot experimental intervention program. The Ministerial Committee for Social and Economic Affairs approved the program outline in August 2012. Funds were allocated to increase public awareness in 50 communities, to carry out experimental environmental intervention activities in 15 communities, to monitor the effectiveness of the intervention activities and for research. Activities under the program started in 2013. Urim, a collective community (kibbutz) located in the Western Negev, was chosen as one of the fifteen communities, following a ZCL outbreak as a result of *L*. *major*. The outbreak started in 2010 with annual reported incidence rate of 103.1/1000 population and continued in 2011 and 2012 with reported incidence rates of 78.4 and 24.7/1000, respectively (Division of Epidemiology, Ministry of Health, Jerusalem, Israel). Many active *M*. *tristrami* burrows were observed on a man-made embankment surrounding the residential area. This observation and the prevailing view that sand flies progress in short hops and do not disperse far from breeding sites [15] led to the assumption that the embankment is the main source of *Leishmania*-bearing *P*. *papatasi* females threatening the residents of Urim. Thus, the original intervention plan was to protect the residential area by clearing the embankment from jirds to create a narrow "cordon sanitaire". Sand fly monitoring was designed to study the spatial and temporal distribution of sand flies and *Leishmania* infections in sand flies and to assess from which directions and what distance sand flies reach the residential area. The monitoring was also intended to verify the likelihood of success of the planned activities against the rodents.

The current work summarizes the sand fly data collected throughout the intensive sand fly surveillance efforts in and around Urim during the summer of 2013. The marking experiment was designed to study the dispersal distances of sand flies by calculating the middle travel distance and not relying only on maximal travel distances. The results have changed the view on the dispersal of sand flies in the area and led to the expansion of the "cordon sanitaire" in which activities against the reservoir should be taken.

## Materials and Methods

### Study site

**Fig 1:**
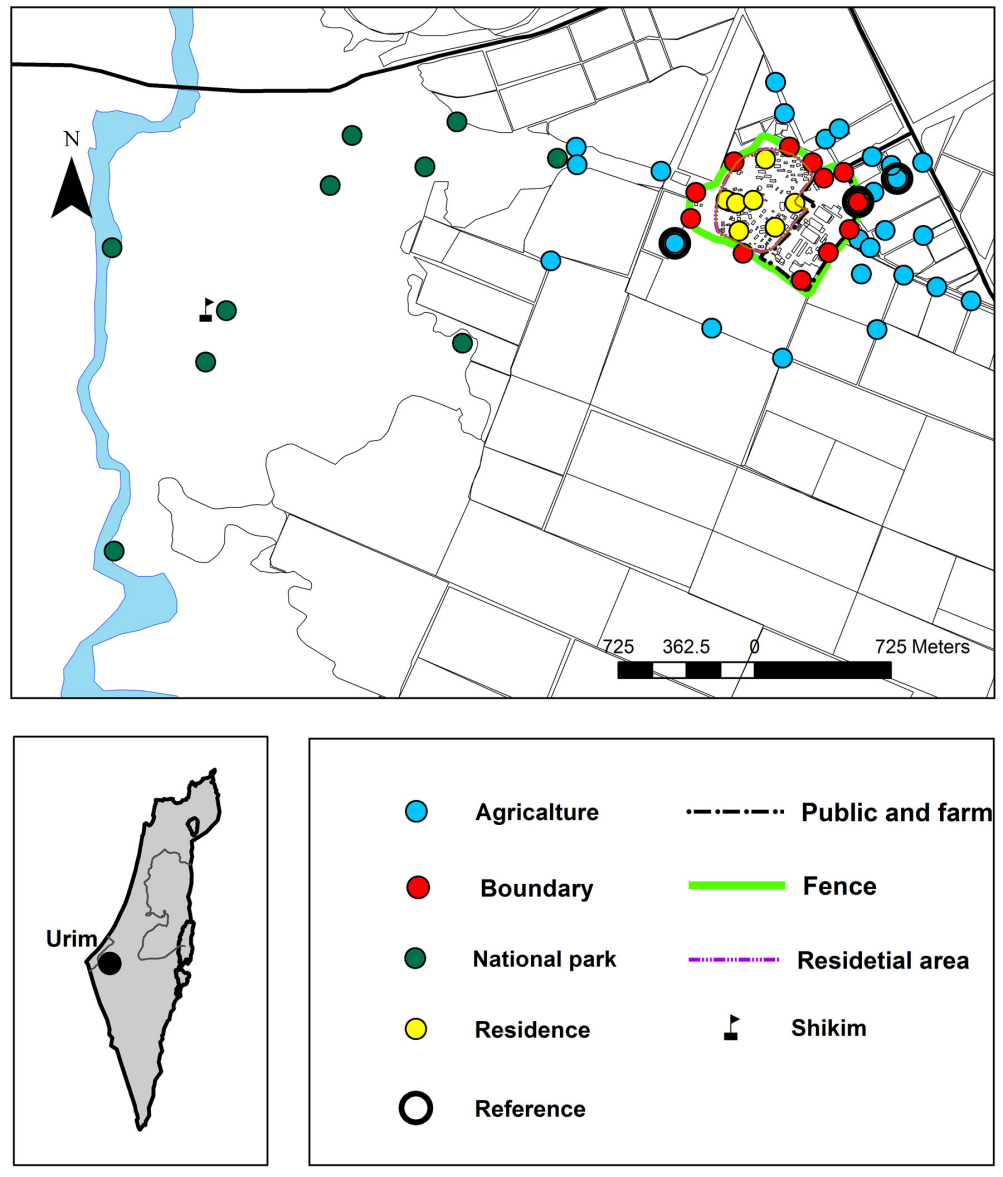
The location of Urim marked on the map of Israel (lower right panel) and a map of Urim and the surroundings showing the study area. Filled circles indicate the trapping sites according to land use categories, black circles indicate the reference traps, lines delineate the zones of Urim.

Urim (31°30N,34°52E) is located in the semi-arid plains of the northwestern Negev of Israel ~25 km west of Be'er Sheva and ~15 km east of the Palestinian Gaza Strip (Fig 1, small map). The summer (May to October) is hot with mean daily temperatures of 32–34°C, often reaching 40°C, and mean night temperatures around 20°C. The coldest months are December to February, with maximum mean daily temperatures of 17°C and mean night temperatures of 7°C. The rainy season is between November and April, mean annual rainfall is around 250 mm. The wind regime in the area is characterized by northwest winds in the late afternoon, replaced by southeast winds during the course of the night [16]. Urim is a small cooperative community (kibbutz) with around 500 residents. The residential area (~0.5km^2^) includes on the west side, small family homes surrounded by communal irrigated gardens (Fig 1, purple line) and on the eastern side public and farm buildings (Fig 1, black line). A boundary zone ~50-100m wide and ~2.6km long (Fig 1, green line) separates the residential area from the surrounding agricultural fields. The boundary zone includes a man-made embankment, ~4m wide at its base and 2-3m high, built to prevent unauthorized movement of vehicles, a dirt road and a high chain-link fence. The agricultural fields around Urim include mainly grain crops such as wheat and barley are watered by rain. Field crops as potatoes, carrots, sunflowers, peanuts and other vegetables are irrigated. Depending on the crop type and crop rotation schemes, some of the plots are not cultivated between the spring harvest and the autumn. A natural recreation area, Eshkol National Park, is located about one km west of Urim. The national park, ~3.5km^2^, is characterized by loess badlands and plains with a landscape cover of biogenic crust matrix and patchy shrub distribution [17]. In some parts of the park, man-made contour banks called *Shikim* [18] were used as a water harvesting system to trap surface runoff water to sustain trees sparsely planted for shade. The National Park is bordered in the east by the agricultural fields of Urim and in the west by the bank of HaBesor stream. Active Tristam's jird burrows were common on the embankment in the perimeter of the Kibbutz. In the farmland, jird burrows were abundant in non-plowed plots and in the narrow wayside by the agricultural roads separating the cultivated fields. Eight out of the twelve (67%) *M*. *tristrami* trapped near the embankment in 2011 and 3 out of 13 (23%) trapped in 2013, were positive by PCR to *L*. *major* (Roni King, Israel Nature and Parks Authority, Jerusalem, Israel). Additional reservoir animal species especially *Psammomys obesus* were not present.

### Sand fly trapping

**Fig 2:**
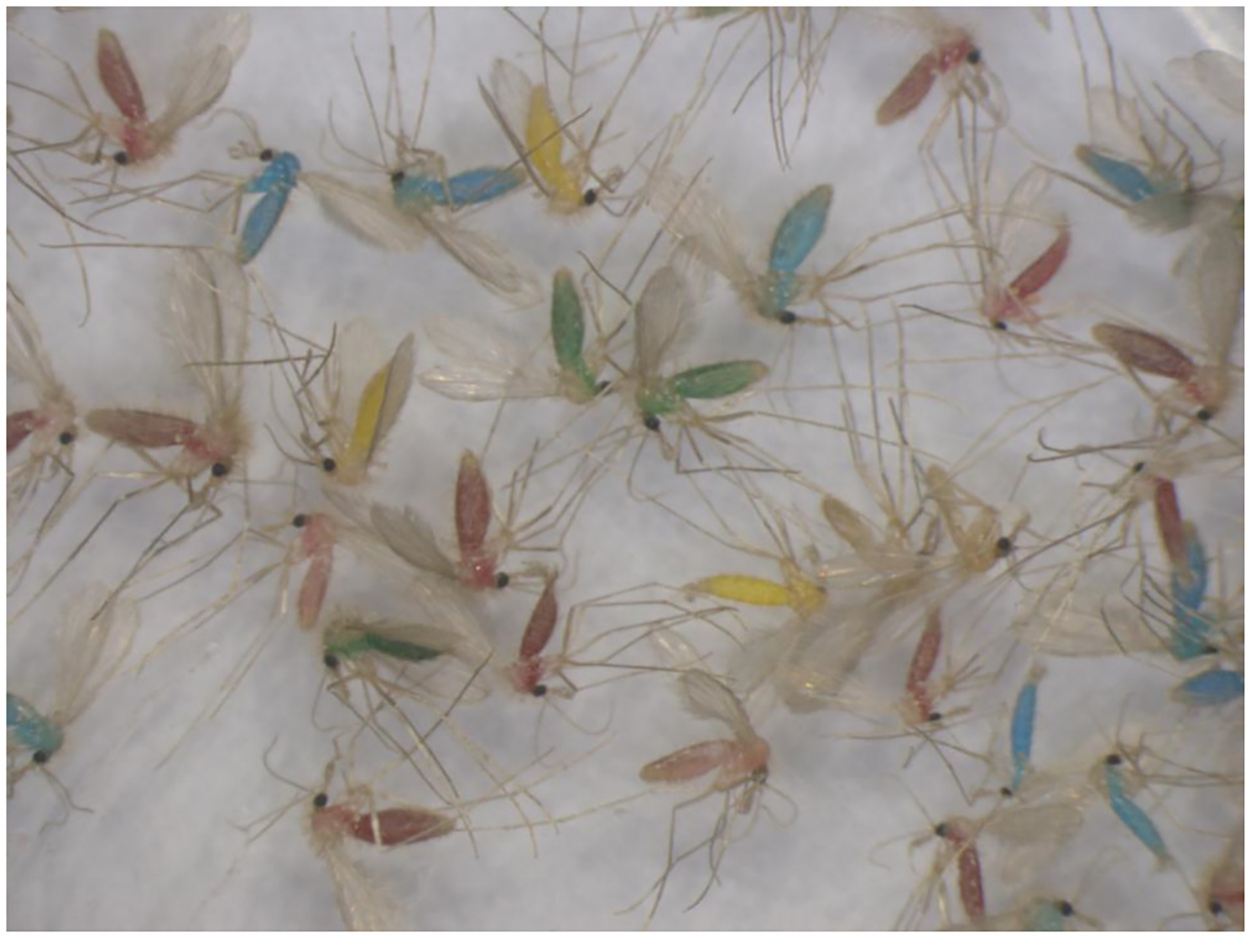
Sand flies marked by feeding on sugar solutions containing food dyes. Marking is demonstrated with *Phlebotomus sergenti* sand flies from the Judean Desert.

Sand flies were collected outdoors between May and December 2013. Modified CDC traps operated without light powered by two 1.2 V AA rechargeable batteries and baited with ~1kg of dry ice, were placed overnight in a vertical position. The openings were parallel to the ground and ~10 cm above it, the fan causing updraft airflow with the collection boxes hanging above the body of the trap. The catch was chilled and kept at -20°C until sorting. All males and samples of females were identified to species. The identification was made by examining the morphology of male genitalia, female spermathecae, and pharynges using the keys of Abonnenc 1972 [19] and Lewis 1982 [20]. In the marking experiment trappings, the numbers of sand flies with visible food dye were noted for each trap and food dye color (Fig 2). The information on the food dyes and the application method is reported in the section "monitoring dispersal distances". In catches exceeding ∼500 specimens, sand fly numbers were estimated by dividing the catch into sub-samples of 1/4-1/8 and counting the sand fly numbers in the sub-sample. All the females from small catches and 100–400 females from traps that collected more than 500 specimens were pooled in groups of up to 20 specimens for molecular detection of *Leishmania* DNA. Engorged females were kept individually for future blood-meal identification. All samples were stored at -20°C until testing.

### *Leishmania* detection

**Fig 3:**
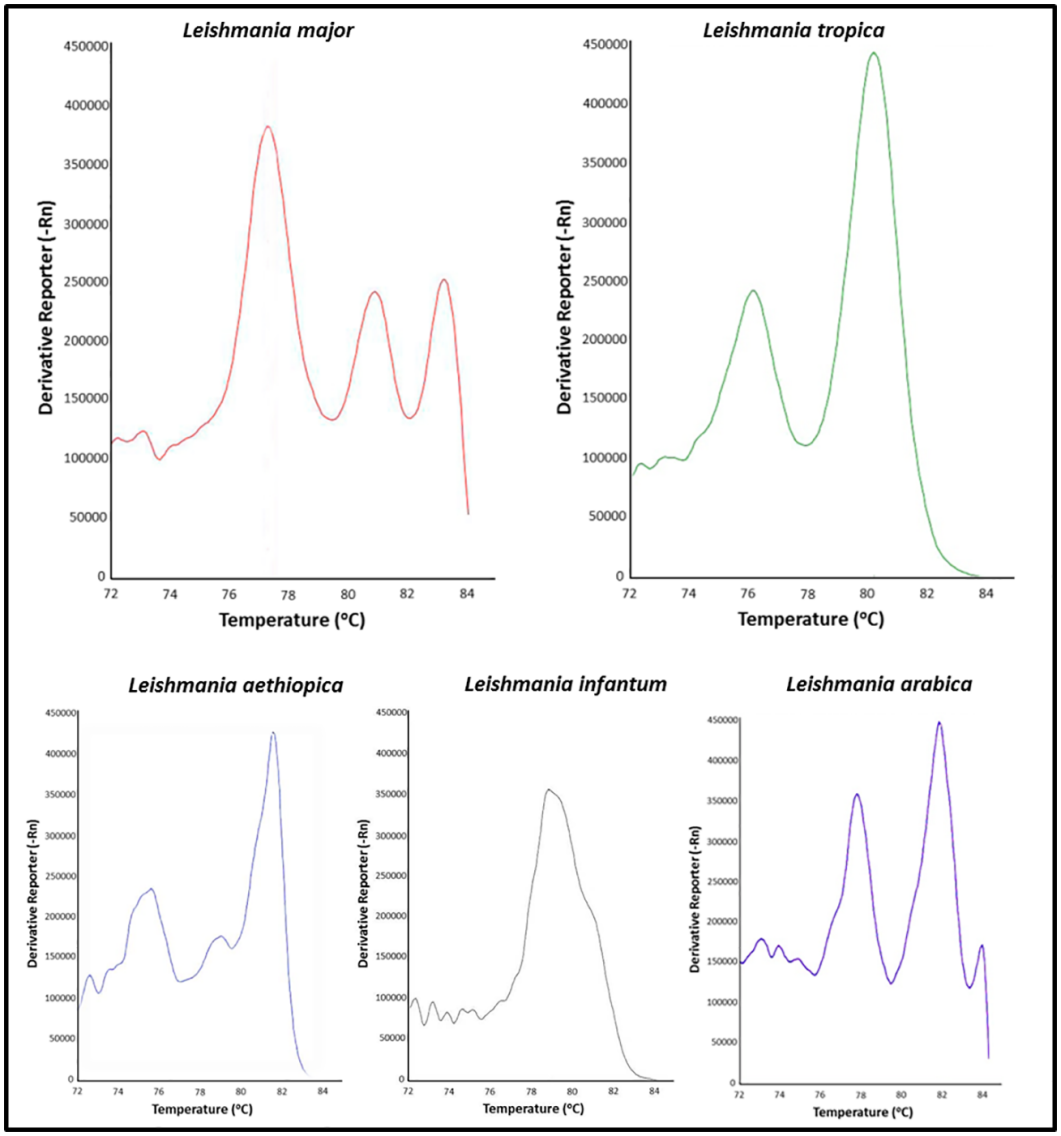
Melting curve features of *Leishmania major*, *L*. *tropica*, *L*. *infantum/donovani*, *L*. *aethipica* and *L*. *arabica*.

DNA was extracted from the pooled intact sand fly females using the PureLink Pro 96 Genomic DNA Kit (Invitrogen by life technology, Carlsbad, CA) following the manufacturer's instructions. To grind the samples, 3mm stainless steel beads and 200μl proteinase K digestion solution were introduced to each tube. Plates of 96 tubes were set in the TissueLyser II (QIAGEN, Valencia, CA). Parameters were set at 30 Hz, 5min. DNA extracts were amplified with the previously described internal transcribed spacer 1 gene (ITS1) primers ITS-219 [19]. The PCR reaction was performed in a total volume of 20μl, containing (10 μl) of AccuMeltHRM SuperMix (Quanta Bioscience, Gaithersburg, USA), 0.5 μM of each primer, 0.1% BSA (w/v), 5% DMSO (w/v) and 3μl DNA, using the 7500 Fast or StepOne Real-Time systems (Applied Biosystems, California, USA). The cycling parameters were 95°C for 5 min; 40 cycles of 95°C for 10 sec; 62°C for 45 sec. Melting curves were generated by the RT-PCR machine software. The florescent signal was measured while raising the temperature to 95°C for 10 seconds, 62°C for 1 minute, and 95°C for 15 seconds (ramp rate, 1%). The high resolution melt analysis (HRM) method described by Talmi-Frank et al. 2010 [21] differentiates between *Leishmania* species. The specificity of the method for the direct identification of *Leishmania* species was further verified in our lab using DNA extracted from *Leishmania* cultures. The cultures used included *L*. *tropica* (MHOM/IL/1990/P283), *L*. *major* (MHOM/PS/1967/Jericho II), *L*. *donovani* (MHOM/SD/1962/1S-CLD2), *L*. *aethiopica* (MHOM/ET/1972/L102) and *L*. *arabica* (MPSA/SA/83/JISH220). The specific melt curves for the five *Leishmania* species are shown in Fig 3. The HRM analysis does not distinguish between *L*. *infantum* and *L*. *donovani* that have almost identical DNA sequence over the amplified 265 bp region [19]. Two samples of *L*. *tropica* and two of *L*. *major* parasites were included in each DNA extraction as standard reference controls. Samples of the positive identifications were validated by sequencing.

### Monitoring the spatial and seasonal distribution

One to three sand fly collections were conducted each month. Traps were set at 55 different sites (Fig 1) in four categories of land use: residential area (8); boundary zone (12); agricultural fields (25); Eshkol National Park (10). Trapping sites were not associated with rodents borrows which were scattered and not clustered. In the residential area the traps were placed in the communal gardens after receiving consent from the Kibbutz Urim Secretariat. We were able to operate up to 16–24 traps each night, thus we were unable to place traps in all sites throughout the season. However, in each trapping night, three traps were set in fixed locations (reference sites—marked by black circles, Fig 1).

### Monitoring dispersal distances

**Fig 4:**
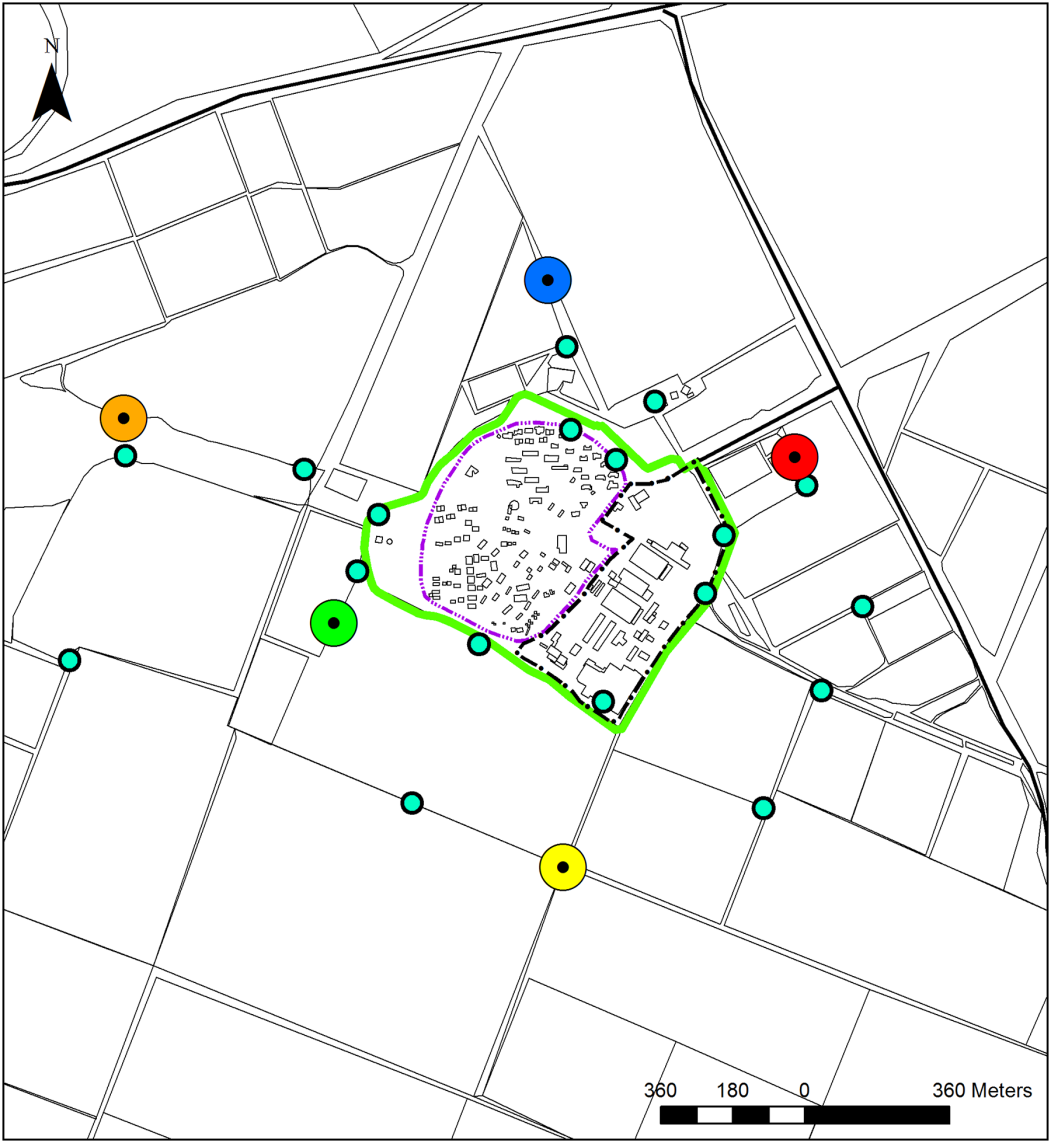
The dispersal experiment set-up. The locations of the marking sites indicated by the large red, blue, orange, yellow and green circles and the monitoring traps indicated by the small turquoise circles.

To study the flight distances of sand flies we used sugar bait marking, exploiting the tendency of sand flies to feed on sugar from the surface of plants [22]. Of the trapping sites we chose five that yielded large captures, each located in a different direction and at a distance of 0.6–1.1 km from the center of the kibbutz (Fig 4). In each site a patch of vegetation ~200 m^2^ was sprayed with 20l of solution containing 10% sugar and 0.5% of one of five different food dyes (Brilliant Blue E133, Red Carmosine E122, Yellow E-102; Orange E110, Brilliant Green E102&E133, Stern, Netanya, Israel). The vegetation was sprayed till runoff using a backpack sprayer (15Lt 425, Solo, Newport News, VA, US). Stubble of wheat ~30cm high dominated the area sprayed with the green sugar solution south-west of the Kibbutz, dead standing annual plants ~30cm high dominated the sites sprayed with the blue (north) red (north-east) and orange (west) sugar solutions, while dead standing annual plants and perennial shrubs of ~50cm high dominated the site sprayed with yellow (south) sugar solutions. The design of the experiment included an attempt to increase the number of marked sand flies by placing two containers each with 1 kg of dry ice without traps, in the middle of each of the five sprayed patches. Using this experimental design, we gave up on the possibility of collecting information on the flying distance of sand flies during one night. In the following two nights we trapped sand flies in 23 locations within a 2km radius of the marking sites: five in the center of each marking site, eight in the boundary zone just inside the surrounding fence and an additional ten in the fields (Fig 4). Marked sand flies in the catch of the 22 distant traps represented the dispersing population. Marked sand flies in the catch of the trap placed in the center of the marking area were considered non-dispersing. The material collected by the five traps placed in the marking sites was used to estimate sand fly densities, male/female ratios, proportions of specimens marked by the color of the site and to calculate the ratios between dispersing sand flies and non-dispersing sand flies.

### Data analysis and statistics

The maximal number of specimens collected in each trapping site was used to show the spatial distribution of sand flies densities. The average size of the catch calculated for each trapping site and category of land use (residential, boundary, agriculture and Eshkol National Park) were used to compare the findings from the four land use categories. Collections of up to 10 specimens were considered small; collections of 11–100 moderate, collections of 101–1000 large and collections of more than 1000 specimens very large. The data from the three reference trapping sites (indicated by black circles in Fig 1), were used to study the seasonal trend.

The level of transmission was monitored by screening "pools" of specimens and calculating the minimal *Leishmania* infection rate (MIR) which assumes that a positive pool contains only a single infected insect [23]. The MIR was calculated as the number of positive pools per 100 females tested. To compare the relative risk that a female sand fly will infect a human with *L*. *major* we calculated the Estimated Risk of Exposure Index (EREI). Following Kilpatrick et al. 2005 [24] and assuming consistent anthropophilic tendency and vector competence for the one vector species present, we calculated a simplified EREI. The EREI was calculated by multiplying the proportion of infected females (number of positive pools divided by the total number of females tested) by the number of female specimens in the relevant catch. The MIR representing the percent of infected females was calculated for each trapping month, trapping site, and land use category. The EREI representing the number of infected sand fly females per trap was calculated for each month and land use category. The EREI representing the relative number of infected sand fly females in the different land use categories, may indicate the relative contribution of each category to the infected sand fly females capable of transmitting *Leishmania* parasites to people.

To calculate the dispersal of sand flies from each of the five marking sites, we used the data of the sand flies marked by the site color from all the traps (22), excluding the one located in the center of the marking site. The trapping area surrounding each of the marking sites was divided into four annuli, separated by 0.5 km (ArcMap 10.2, Esri, Redlands, CA, US). The mean distance traveled (MDT) was calculated following the method developed by Lillie et al. 1981 [25] and White & Morris 1985 [26]. To account for differences in trap densities a correction factor (CF) was calculated for each annulus. CF = (the area of the annulus × No. of traps per annulus) / area of trapping. The estimated recapture (ER) was calculated for each annulus. ER = (No. of colored sandflies per annulus × CF) / No. of traps per annulus. The mean traveled distance was calculated using the formula: MDT = Σ (ER × median distance (for each annulus)) / Total number of ER). MDTs were calculated separately to evaluate the dispersal of females and males from each of the marking patches. The General MDT calculation was done separately for females and males.

One-way ANOVA and post hoc Tukey's HSD tests were used to analyze the effect of land use category on sand fly densities. A Kruskal-Wallis test was conducted to test the difference in sex ratio between the land use categories. Sand fly numbers and sex ratios were Box-Cox transformed to meet statistical tests assumptions. Correlation analysis was conducted between total catch per trap and male/female ratio. A difference test for proportion was conducted between every two sites to detect differences in the proportion of marked sand flies and the marking of females and males between the five marking sites. Bonferroni corrections were applied to prevent false positive results caused by multiple comparisons. Correlation analysis was conducted between the non-dispersing sand flies and the dispersing sand flies. Chi-square was used to test whether the ratios between dispersing and non-dispersing sand flies differ between males and females. STATISTICA software, version 12.6 (StatSoft Inc., Tulsa, Oklahoma, USA) was used for all the statistical analysis.

## Results

### Surveillance data

A total of 122,507 specimens were collected (females comprising 68%), during 14 trapping nights, using a total of 189 traps sampling 55 sites. Catch size varied between 0–3755, male/female sex ratios in catches of n≥5 were between 0–1.48. No correlation was found between the sex ratio and the catch size (r_(46)_ = 0.27, p = 0.065). The very few *Sergentomyia* specimens were discarded. *Phlebotomus papatasi* was the sole *Phlebotomus* species found in the trappings. All the male specimens had coxite with very small process near the base, long and cylindrical style with three short terminal spines and two basal spines much closer to each other than to the terminal spines and surstyle with two short spines at the end. The females were characterized by pharyngeal armature of scaly teethes arranged into a wide meshed network restricted to the posterior third of pharynx. The spermatheca were cylindrical with 8–12 rings (apical segment short) and the relative lengths of the ascoids on antennal segment IV were less than 0.6 [27]. Approximately 20% of the females (15,023) were tested for the presence of *Leishmania* DNA by PCR. *Leishmania* DNA was detected in 78/807 pools (~10%), in 30/55 sites sampled. The MIR was 0.51. The melting curves of all the positive samples from Urim were similar to those of the *L*. *major* control samples (Fig 3), while *L*. *tropica* and *L*. *infantum* melting curves were identified in simultaneously tested samples from other regions of Israel.

### Seasonal trends

**Fig 5:**
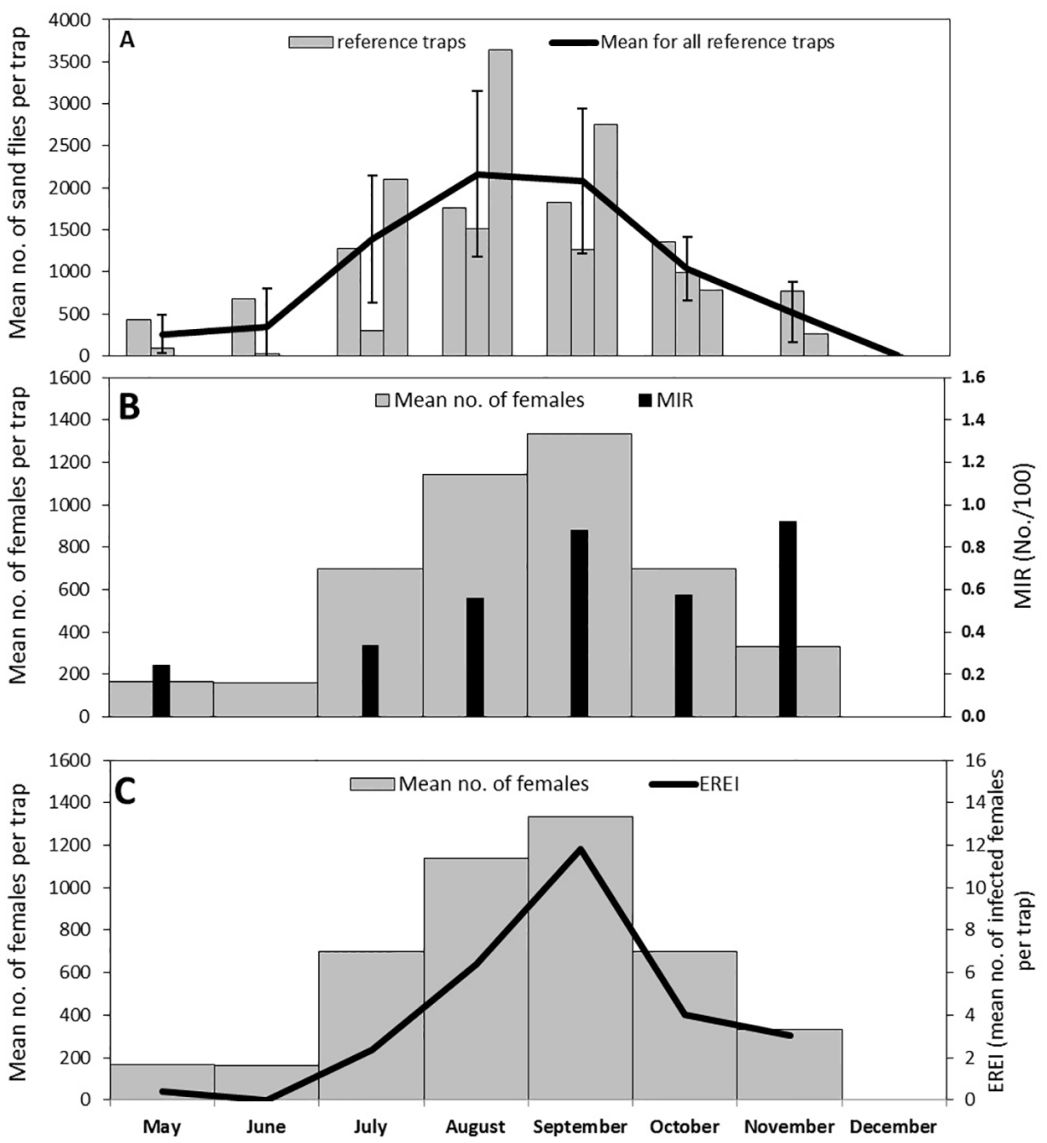
The seasonal distribution of *Phlebotomus papatasi* based on the reference traps data. A. population densities; B. female densities and MIR; C. female densities and EREI.

The seasonal trend observed was generally similar in the three reference traps (Fig 5A). Sand fly numbers were low in May and June, increased in July peaked in August and September reaching between 1,500 and 3,500 specimens per trap. Numbers started to decline in October and further declined in November. Traps operated in December collected no sand flies. *Leishmania major* DNA was detected in pooled sand fly females from all the collection nights, except the collection night in June. *Leishmania* MIR was relatively low in May and July, increased in August and was high in September and November (Fig 5B). The highest estimated risk of exposure EREI, was in September (Fig 5C), EREI was very low in May & June and moderate in July, August, October & November.

### Spatial distribution

**Fig 6:**
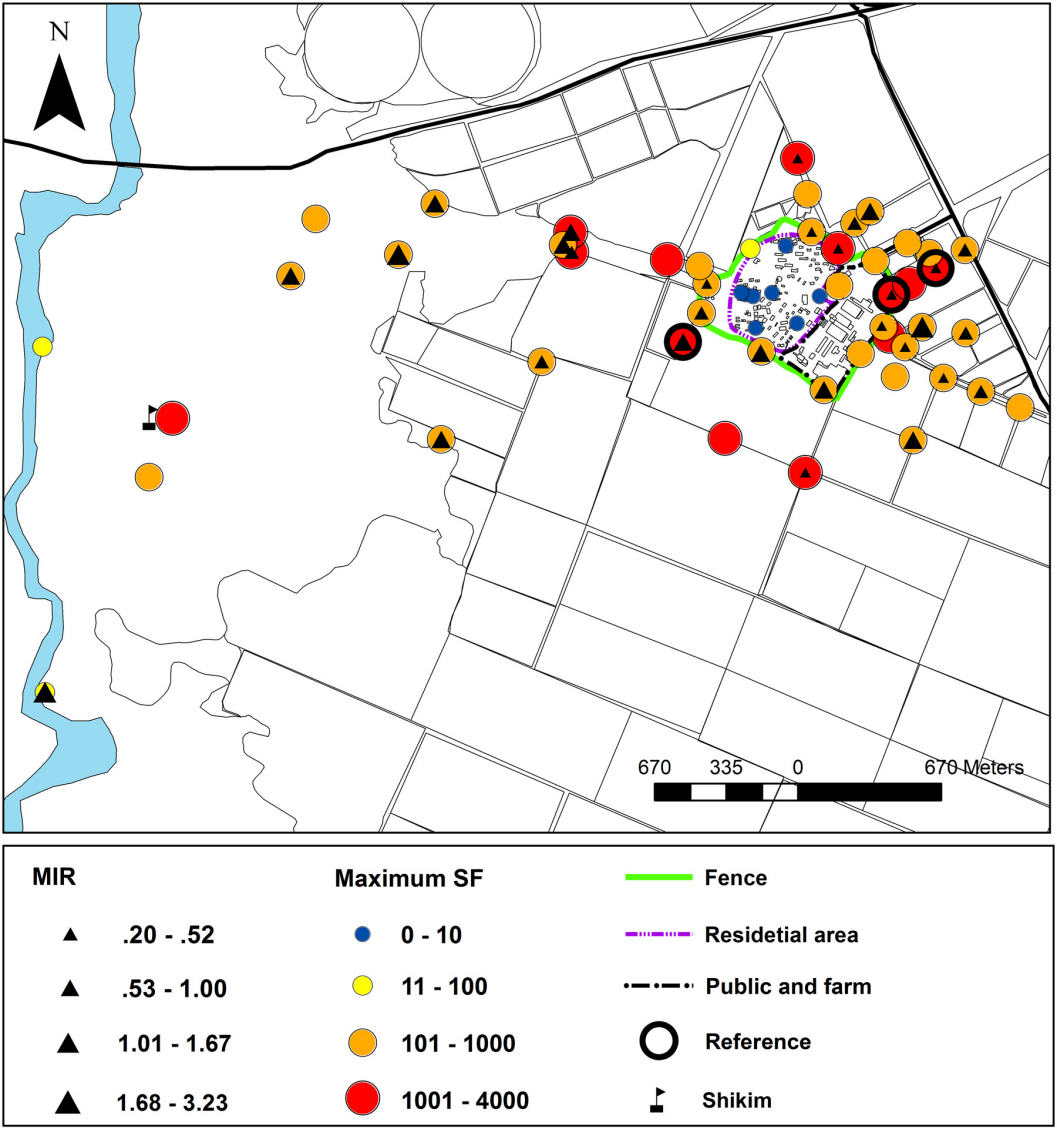
The spatial distribution of sand flies and infection rates in each trapping site. Maximal number of sand flies expressed by the size and color of the circles and the MIR values expressed by the size of the black triangles.

The maximal number of sand flies collected in each trapping site and the MIR are presented in Fig 6. The trapping efforts and the sand fly data according to land use categories are summarized in Table 1. Traps placed in the area used for residence collected only a few specimens (0–5) and most were females (82%). In the boundary zone close to the embankment, females comprised 69%. In most sites (9/12), the catch was large (114–946 specimens). A high proportion of females (86%) and large differences between the trapping sites characterized the sand fly catch in the Eshkol National Park. Catches were moderate in the two trapping sites located on the bank of HaBesor stream, large in seven sites, and very large in a site (marked by a flag in Figs 1 and 6) located amid a *Shikim* system. Catches were generally large to very large in the agricultural fields and females comprised 67%. The differences between the number of specimens trapped in the four land use categories were significantly different (F_(3,183)_ = 80.6, p<0.01). Post hoc analysis showed significant differences between the land use categories, except between the boundary zone and the Eshkol National Park. Sand fly densities were similar in all directions around Urim, there was no direction with notably higher or lower densities (Fig 6). The male/female sex ratio differed between the land use categories (H_(2,173)_ = 26.63, p<0.01). Significant difference in sex ratio was found between the Eshkol National Park and both agriculture and boundary areas. On the other hand, no significant difference was found between the agricultural and boundary areas (Table 1). *Leishmania major* DNA was not detected in females collected in the residential area. *Leishmania* MIR was similar in females collected in the boundary zone (0.43) and the agricultural fields (0.5). The MIR was highest (1.12) in females from the Eshkol National Park area. The EREI indicating the relative contribution of infected sand fly females based on densities and MIR was higher in the Eshkol National Park and the agricultural fields in comparison with the boundary zone (Table 1).

**Table 1:** Trapping efforts and sand fly data summarized according to land use category.

Land use category	No. of sites	No. of trap nights	Mean no. of sand flies/trap ± se*	Mean male/female ratio ± se*	No of females analyzed/no. of positive pools	MIR	EREI
Residence	8	15	1±0.35^a^	**	51/0	0.00	0
Boundary	12	73	464±57^b^	0.52±0.04^a^	6678/29	0.43	1.19
Agriculture	25	84	981±99^c^	0.49±0.03^a^	7040/35	0.50	3.39
Eshkol National Park	10	17	360±96^b^	0.17±0.2^b^	1254/14	1.12	3.46
Total	55	189			15023/78	0.51	

* Statistically significant differences (post hoc analysis) are marked with different letters.

** Male/Female ratio was not calculated since all traps collected less than 5 specimens.

### Dispersal

The total number of sand flies captured during the two trapping nights following the spraying of sugar solutions containing food dyes was 38,958. The five traps placed in the marking sites collected 1,111–5,853 specimens; females comprised 60–87% (Table 2). The proportion of sand flies that contained visible traces of the site's marking solutions were similar in the material collected in four (green, blue, red and orange) of the five marking sites. In these collections the marking rates of the males (23–30%) were significantly higher than those of the females (8–15%) (red Z = -2.96, p = 0.0015; green, blue, orange Z = -3.896, p<0.00001). Marking rates in the catch from the fifth site (yellow) were significantly higher (Z = -3.896, p<0.01 after Bonferroni correction). The marking rates of the males (52%) were slightly higher than those of the females (46%). The difference was not significant (Z = -2.1, p = 0.143) (Table 2).

**Table 2:** Number and proportion of marked non-dispersing sand flies, in the marking sites traps.

Color of marking solution	Females	Males
	No./Marked (% marked)	No./Marked (% marked)
Green	1733 / 138 (8)	253 / 59 (23)
Red	440 / 103 (15)	671 /106 (24)
Yellow	2055 / 947 (46)	395 /206 (52)
Orange	4351 / 508 (12)	1502 /358 (24)
Blue	1105 / 114 (10)	434 /130 (30)
Total	9915 /1810 (18)	3024 /859 (28)

**Fig 7:**
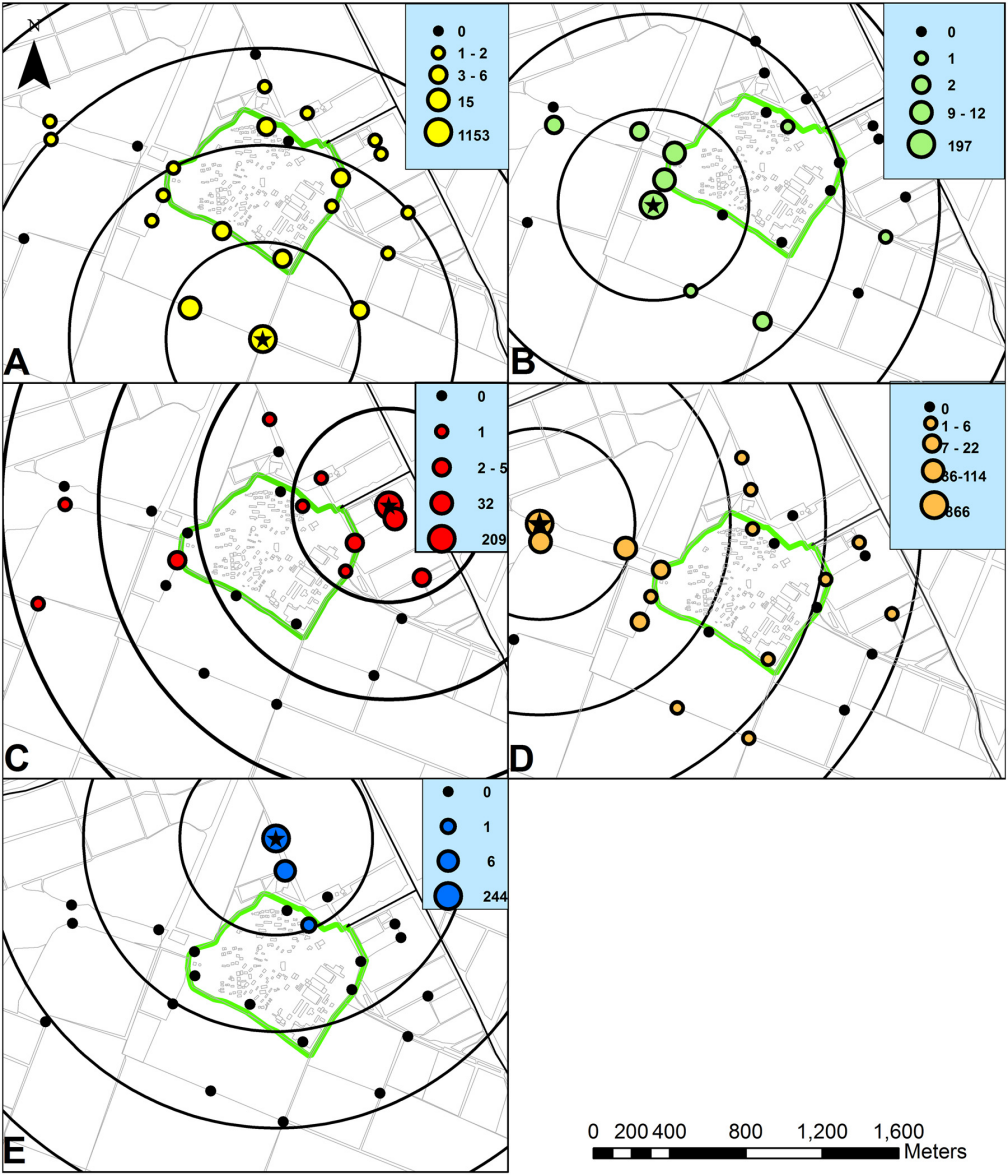
Sand fly dispersal from the five marking sites. A-yellow, B-green, C-red, D-orange, E-blue. Black stars indicate the marking sites, the annuli used for MDT calculation are marked by the concentric rings.

The distribution of marked sand flies captured in each of the trapping sites, and the annuli used for MDT calculations are presented for each marking color in Fig 7. The data of the marked sand flies collected by the 22 distant traps, the maximal travel distances and the calculated middle travel distances (MDTs) for the females and the males are summarized in Table 3. The number of dispersing sand flies (Table 3) was positively correlated with the number of the corresponding non-dispersing sand flies (Table 2) (females r_(3)_ = 0.56, p<0.05; males r_(3)_ = 0.35, p<0.05). Dispersing green sand flies were found in 8/22 traps, blue specimens were found in 2/22, red in 10/22 yellow in 18/22 and orange in 12/22. Apart from the blue marking, dispersing sand flies were found in traps placed on the other side of the residential area. For all marking sites, the maximal flight distances were longer for females (0.48–1.91 km) than for males (0.18–1.53 km). For each of the marking colors, marked males were not caught in the corresponding most distant trap. Female sand flies marked by the red, yellow and orange food dyes were found in the collection sites furthest away from the corresponding marking site (1.88km, 1.57km and 1.91km). Females marked by the green color were found at a distance of 1.23km while the most distant trap was about 90 m further away (1.32km). The most distant trap from the blue marking site was at 1.52km. However, the very few blue marked sand flies retrieved were found in only two very close traps (Fig 7E). Males marked by all food dyes except the blue and females marked by the green and orange food dyes were collected on the first trapping night (second night after marking) in the maximal distance. Females marked by the red and yellow food dyes were collected on the first trapping night very close to the maximal distance. The ratios between dispersing to non-dispersing females were greater (χ^2^_(4)_ = 102.64, p<0.001) than the ratios between dispersing and non-dispersing males (Table 4). The calculated MDTs were 0.25–0.95km for females and 0.25–0.93 for males (Table 3). The presence of only one blue male obviated the calculation of male-MDT for the blue marking. The calculated MDTs for females were slightly greater than those of the males for all the marking colors except the orange. The calculated overall MDT for females (0.74km) was somewhat smaller than the general overall MDT calculated for the males (0.82km) (Table 3). The overall MDT calculated for the females and males together was 0.75km.

**Table 3:** The number of dispersing sand flies, mean (MDT) and maximal (Max) travel distances.

Marking color	No. of marked sand flies collected by the distant traps		Distance (km)			
			Females		Males	
	Females	Males	MDT	Max	MDT	Max
Green	24	6	**0.58**	1.23	**0.25**	0.49
Red	32	19	**0.78**	1.88	**0.77**	1.13
Yellow	43	7	**0.95**	1.57	**0.92**	1.18
Orange	184	22	**0.69**	1.91	**0.93**	1.53
Blue	6	1	**0.25**	0.48	*	0.18
**Total**	**289**	**55**	**0.74**		**0.82**	

* Not calculated due to a sample of only one specimen.

**Table 4:** The ratios between dispersing and non-dispersing sand flies.

Marking color	Ratios between dispersing to non-dispersing sand flies	
	Females	Males
Green	0.17	0.10
Red	0.31	0.01
Yellow	0.05	0.18
Orange	0.36	0.03
Blue	0.05	0.06
**Total**	**0.16**	**0.06**

## Discussion

*Phlebotomus papatasi*, the vector of *L*. *major* in Israel, was the sole *Phlebotomus* species found in the collections. *P*. *papatasi* was the only human biting sand fly species in collections from the Central Negev ~45 km south of Urim [3,28] and the Jordan Valley near the Dead Sea area in Israel [29,30], as well as in collections from Northern Sinai Desert of Egypt [31,32] and West Alexandria Egypt [33]. The large number of specimens characterizing most of the catches indicated the presence of large populations of sand flies. Sand flies were abundant throughout the long summer showing one seasonal peak in the warmest months, August and September (Fig 5A). Unimodal seasonal pattern of *P*. *papatasi* showing a seasonal peak in September was reported from Iran [34]. However, in most studies seasonal pattern of *P*. *papatasi* was bimodal. Several studies reported a large early peak followed by a second smaller one in September or October [32,35,36]. In other studies, the first peak in June was smaller and the second one in August or September was larger [37,38]. Variations in the seasonal patterns of *P*. *papatasi* were observed between successive years in the same location [39] and between locations in the same year [40,41]. The accumulating data demonstrate flexible seasonal pattern of *P*. *papatasi* subjected to modulation by weather variables and other environmental factors.

*Leishmania major* DNA was detected by PCR in females throughout the season except in June (Fig 5B). Minimal infection rates (MIR) were low in early season, increased towards the second half of the summer, in parallel to the increase in sand fly densities. MIR remained high in the autumn until the end of the season, while sand fly densities decreased. Infected *P*. *papatasi* females were present throughout most of the activity season; however the risk of infection was highest in September (Fig 5B and 5C). Our results are in accord with previous studies that found higher *L*. major infection rates in *P*. *papatasi* females, in the second half of the sand fly season. In Sinai, Egypt, the highest infection rate was recorded in July in correlation with the highest sand fly human landing density [31]. In additional study from Sinai, infections rates of female *P*. *papatasi* increased towards the end of the summer and were correlated with the proportion of gravid females [32]. A study from the southern Jordan Valley of Jordan, reported the presence of infected females form June until November with highest infection rates occurring in November [42]. In Iran infected females were found from May until October and highest infection rates in mid-September coincided with the large second peak of sand fly abundance [43].

Taking into account an incubation period of around two to three months for zoonotic cutaneous leishmaniasis as a result of *L*. *major* [44] the EREI predicted a seasonal peak to the onset of morbidity in November and December. Given an additional period of several weeks to consult a doctor and reach a diagnosis, there is a full accordance between the seasonal peak of positive laboratory human cutaneous leishmaniasis diagnosis in December and January as reported by Ben-Shimol et al. 2015 [14] and the EREI prediction. Early winter peaks in the monthly distribution of zoonotic human leishmaniais caused by *L*. *major* were previously reported from Jordan and Tunisia [37,45].

### Spatial distribution

The residence area of Urim is a small "island" of homes and cultivated garden surrounded by a "sea" of farmland dominated by annual crops. Only a few *P*. *papatasi*, none infected with *Leishmania* parasites, were captured in traps placed in the residential area between the homes, while sand flies were abundant in traps placed in the surrounding boundary area and the agricultural fields (Table 1, Fig 6). Similar results, i.e. small number of sand flies in traps placed in residential areas and larger numbers in traps placed in neighboring fallow and cultivated areas, were reported from the southern Jordan Valley [29], the Judean Desert [46] and the central Jordan Valley [47,11]. *Phlebotomus papatasi* is endophilic and anthropophilic species [6]. Therefore, even the low densities observed in traps placed in the built-up areas, were sufficient to cause severe biting nuisance to the residents of Urim throughout the warm season.

Sand fly catches in the boundary zone were significantly larger than catches in the residential area but smaller than catches in the fields. The larger catches in the surrounding fields and the relatively small size of the boundary zone compared with the size of the field area do not support the hypothesis that the man-made embankment was the main source of *Leishmania*-bearing sand fly threat to the residents of Urim.

The large numbers of sand flies collected in most trapping sites in the agricultural fields, in all directions and at all distances, indicate the existence of many scattered sources of sand flies. The agricultural environment around Urim in the summer and autumn was a complex patchwork of irrigated and non-irrigated plots in various stages of cultivation separated by agricultural roads with non-cultivated narrow waysides. This environment seemed to offer *P*. *papatasi* many niches suitable for development and resting. Development could occur in rodent burrows that were abundant in the non-cultivated plots and waysides. In addition, the deep and relatively cool and humid cracks with cut roots and branches between the large soil clods created by plowing might have contributed suitable breeding sites for *P*. *papatasi* regardless of rodent burrows. The potential role of a plowed field as a major breeding source for *P*. *papatasi* was noted in studies from the Jordan Valley and Dead Sea areas in Israel [30,48]. Positive association between agriculture and irrigation and a high abundance of *P*. *papatasi* was described in studies from Jordan [42] and Israel [29]. A different situation was reported from Tunisia. While irrigation had a positive effect on the abundance of the vectors of *L*. *infantum*, *P*. *papatasi* densities were positively correlated with aridity and the species was more abundant in the non-irrigated areas [37, 49].

In the Eshkol National Park, differences in sand fly catch size between trapping sites were large (Table 1, Fig 6). Sand fly catches were very large only in one site the *Shikim* (marked by a flag in Figs 1 and 6). Probably, as in the agricultural fields, higher soil moisture can account for the increase carrying capacity of this specific site for sand fly development. *Leishmania* infection rates were highest in females captured in the Eshkol National Park (Table 1) indicating a strong association between the vectors and the reservoir animals. Strong association between *P*. *papatasi* and rodents in arid and semi-arid natural environments was reported in earlier studies [50]. Low soil moisture was considered the major factor limiting *P*. *papatasi* development [3,40]. In dry environments, sand fly development mainly occurs in rodent burrows, which provide microhabitats suitable for the development of sand flies [3, 48].

The lower MIR of the females collected from the agricultural fields (0.5) compared with the high MIR in the Eshkol National Park (1.12) indicate a weaker association between sand flies and rodents. Given the great density of *P*. *papatasi* in the agricultural fields, even with relatively low MIR, the number of infective females was rather large as indicated by the risk of exposure (EREI).

### Dispersal

Spraying the vegetation with sugar solutions containing food dyes has the advantage of marking sand flies in their habitat without interrupting their normal behavior by capture, treatment and release [22]. Additional advantages are the ability to mark a large number of individuals when sand fly densities are high, and the ease with which marked specimens can be separated under a stereoscopic microscope (Fig 2). The proportions of marked males were higher than the proportions of marked females in the marking sites traps. Schlein 1987 [22] reported similar marking rates of males and females. Marking rates were significantly higher in the southern marking site where sugar solution containing yellow food dye was sprayed on dead annual plants and perennial shrubs (Table 2). The vegetation in the other four marking sites was lower, sparser and did not include perennial shrubs. Higher feeding rates on denser vegetation and plants that possibly attract sand flies [22] could be expected.

The dispersal of sand flies from four out of the five marking sites (green, red, yellow and orange) had similar characteristics, in terms of travel distances and distribution. Sand flies marked by these food dyes were found in trapping sites on the other side of Urim and in different directions (Fig 7A–7D). The capture of only a small number of "blue" specimens outside the marking site (Fig 7E, Table 3) was exceptional. This result may reflect the behavior of the local sand fly population around the blue marking site north of Urim, but more likely the result is atypical. The movement of sand flies in all directions and to distances greater than 1km indicated that sugar fed sand flies in the agricultural fields around Urim were remarkably mobile. The lack of clear movement along the wind direction axis northwest—southeast (northwest in the first part of the night and southeast in the second part [16]), indicates that the dispersal of *P*. *papatasi* was not assisted by wind. No evidence for wind assisted dispersal, and a high mobility of sand flies were reported for *P*. *ariasi* in south of France [51].

The maximal dispersal distance recorded in the present study was 1.91km for females and 1.53km for males. Marked females were found in the furthest trapping site in three cases and at a distance only slightly shorter than the furthest trap, in the fourth case (Table 3). As the maximal dispersal range recorded for females in our study was limited by the distances between the marking sites and the most distant traps it is safe to assume that the actual flight range of females was longer. Marked males were not found in the furthest traps in any of the cases. Hence, the results seem to reflect the actual maximal travel distances of male *P*. *papatasi*. Yuval et al. 1988 [30] found dispersing *P*. *papatasi* in a fallow field at a distance of 400m away from the closest *Psammomys obesus* burrows. The authors concluded that the dispersal was mainly a female activity, and that dispersal of infected females away from the site of infection may result in transmission of leishmaniasis at considerable distance from the sites of vector reservoir contact. Alexander and Young 1992 [52] cited a publication of Streklova and Kruglov from 1985 reporting a flight distance of up to 4 km for *P*. *papatasi* in Uzbekistan. Killick-Kendrick et al. 1984 [51] mentioned that researchers in the Soviet Union concluded that sand flies (probably *P*. *papatasi)* occasionally travel a distance of 1.15km. Maximal travel distance of females *P*. *ariasi* in the south of France were 2.2 km when the record for males was 0.6km [51]. Additional studies on the dispersal of sand flies in the Old World found maximal travel distances of 730m for *P*. *orientalis* Parrot, 1936 in Sudan [53] and 289 m for P. *longipes* Parrot and Martin, 1939 in Ethiopia [54].

The maximal travel distance provides information on the distance a sand fly can reach, but do not provide information on the typical travel distance of the sand fly population. Estimation of the distance at which sufficient numbers of the sand fly population travel and the flight range relevant for epidemiology, disease transmission and control, can be derived from the MDT. The MDT value calculated for *P*. *papatasi* around Urim was 0.75km. The MDT values for the females were slightly larger or similar to the MDT values calculated for the males in three cases (green, red and yellow) and somewhat smaller in the fourth case (orange). The overall MDTs for the females (0.74km) and for the males (0.82km) were similar. The slightly lower overall MDT calculated for the females was affected by the relatively large number of females marked by the orange food dye and probably do not represent a shorter effective dispersal distance of females. The ratios between dispersing and non-dispersing sand flies were used to compare the dispersing tendency of the females and the males. The higher values observed for females for each of the marking colors imply a greater tendency of females to disperse. The results of the maximal travel distance and the ratios between dispersing to non-dispersing sand flies indicate that females tend to disperse more than males and travel longer distances. Nevertheless the similar MDT results for females and males and a maximal distance of more than 1.5km recorded for males suggest that males and not only females disperse, and that males, although tend to disperse somewhat less than females, do not necessarily stay close to breeding sites as indicated in the literature [29,30, 42,51, 55–57].

### Summary and conclusions

Marking sand flies in the field using sugar solutions containing food dyes and an integrated methodical monitoring scheme, we were able to collect in a short period of several months, epidemiologically meaningful information. The overall results of the present work indicated the existence of dense and mobile sand fly population around Urim. Scattered over large areas in the agricultural fields there seemed to be numerous development sources and suitable resting sites for sand flies. Sand flies apparently moved in all directions to distances typically of at least 0.75km. The risk of exposure to *Leishmania*-infected sand fly female had a distinct seasonal peak in September.

Given the current study results, the "cordon sanitaire", in which actions against the reservoir rodents had to be carried out, was extended to the agricultural fields. Identifying a season-high risk of infection in September could be used to encourage the residents to enhance the use of personal protection measures at the critical time.

The travel distance noted in the current work did not fit the widely-held view according to which sand flies stay near breeding sites and have a short limited flight range. It is possible that the favorable environment of the agricultural fields providing many moist microsites suitable for resting, allowed the optimal expression of the dispersal capacity noted in our study. In addition, the good energetic state of the sugar fed sand flies in our experiment might have contributed to the long travel distances observed. Perhaps in natural habitats and/or when conditions are less favorable the full dispersal capacity is not expressed and travel distances are shorter. We hope that the great importance of the dispersal distances of sand flies to epidemiology and control and the simple marking method used, will lead to additional studies and the consolidation of data-driven hypotheses regarding the effective flight range of medically important sand fly species and the factors affecting it.

## References

[1] Jaffe CL, Baneth G, Abdeen ZA, Schlein Y, Warburg A. Leishmaniasis in Israel and the Palestinian Authority. Trends Parasitol. 2004; 20:328–332. 15193564 10.1016/j.pt.2004.05.001

[2] Schlein Y, Warburg A, Schnur LF, Gunders AE. Leishmaniasis in the Jordan Valley II. Sandflies and transmission in the central endemic area. Trans R Soc Trop Med Hyg. 1982; 76:582–586. 6758219 10.1016/0035-9203(82)90215-2

[3] Schlein Y, Warburg A, Schnur LF, Le Blancq SM, Gunders AE. Leishmaniasis in Israel: reservoir hosts, sandfly vectors and leishmanial strains in the Negev, Central Arava and along the Dead Sea. Trans R Soc Trop Med Hyg. 1984; 78:480–484. 6385358 10.1016/0035-9203(84)90067-1

[4] Anis E, Levental A, Elkana Y, Wilamowski A, Pener H. Cutaneous leishmaniasis in Israel in the era of changing environment. Public Health Rev. 2001; 29:37–47. 11780715

[5] Jacobson RL, Eisenberger CL, Svobodova M, Baneth G, Sztern J, Cavalho J, et al Outbreak of cutaneous leishmaniasis in Northern Israel. J Infec Dis. 2003; 188:1065–1073. 14513429 10.1086/378204

[6] Schnur LF, Nasereddin A, Eisenberger CL, Jaffe CL, El Fari M, Azmi K, et al Multifarious characterization of Leishmania tropica from a Judean desert focus, exposing intraspecific diversity and incriminating *Phlebotomus sergenti* as its vector. Am J Trop Med Hyg 2004; 70(4):364–72. 15100448

[7] Svobodova M, Votypka J, Peckova J, Dvorak V, Nasereddin A, Baneth G, et al Distinct transmission cycles of *Leishmania tropica* in 2 adjacent foci, Northern Israel. Emerg Infect Dis. 2006; 12:1860–1868. 17326936 10.3201/eid1212.060497 PMC3291354

[8] Gunders AE, Foner A, Montilio B. Identification of *Leishmania* species isolated from rodents in Israel. Nature. 1968a; 219:85–86. 5659632 10.1038/219085a0

[9] Gunders AE, Lidror R, Montilo B, Amitai P. Isolation of *Leishmania* sp. from *Psammomys obesus* in Judea. Trans R Soc Trop Med Hyg. 1968b; 62: 465.

[10] Wasserberg G, Abramsky Z, Anders G, El Fari M, Schoenian G, Schnur L, et al The ecology of cutaneous leishmaniasis in Nizzana, Israel: infection patterns in the reservoir host, and epidemiological implications. Int J Parasitol. 2002; 32:133–143. 11812490 10.1016/s0020-7519(01)00326-5

[11] Faiman R, Abbasi I, Jaffe CL, Motro Y, Nasereddin A, Schnur LF, et al A newly emerged cutaneous leishmaniasis focus in Northern Israel and two new reservoir hosts of *Leishmania major*. PLoS Negl Trop Dis. 2013; 7(2): e2058 10.1371/journal.pntd.0002058 23437408 PMC3578753

[12] Talmi-Frank D, Jaffe CL, Nasereddin A, Warburg A, King R, Svobodova M, et al *Leishmania tropica* in rock hyraxes (*Procavia capensis*) in a focus of human cutaneous leishmaniasis. Am J Trop Med Hyg. 2010a; 82(5): 814–818. 10.4269/ajtmh.2010.09-0513 20439960 PMC2861385

[13] Gandacu D, Glazer Y, Anis E, Karakis I, Warshavsky B, Slater P, et al Resurgence of cutaneous leishmaniasis in Israel, 2001–2012. Emerg Infect Di. 2014; 20(10): 1605–1611. 10.3201/eid2010.140182 PMC4193169 25271882

[14] Ben-Shimol S, Sagi O, Codish S, Novack V, Barrett C, Fruchtman Y et al Dramatic increase in laboratory-diagnosed human cutaneous leishmaniasis cases in southern Israel 2007–2013. Infec Diseas 2015; 47:161–167. 10.3109/00365548.2014.977342 25622937

[15] Munstermann LE. Phlebotomine sand flies, the Psychodidae In: Marquardt WC, Black WC, Freier JE, Hagedorn HH, Hemingway J, Higgs S, James AA, Kondratieff B, Moore CG, editors. *Biology of Disease Vectors*, 2nd ed San Diego: Elsevier 2004 pp. 141–151.

[16] Israel Meteorological Service; 2015. Accessed: http://www.ims.gov.il/IMS/CLIMATE/LongTermInfo/

[17] Golodets C, Boeken B. 2006 Moderate sheep grazing in semiarid shrubland alters small-scale soil surface structure and patch properties. Catena 2006; 65: 285–291.

[18] Eldridge DJ, Zaady E, Shachak M. Microphytic crusts, shrub patches and water harvesting in the Negev Desert: the *Shikim* system. Landsc Ecol 2002; 17:587–597.

[19] Abonnec E, Les Phlébotomes de la région éthiopienne (Diptera, Psychodidae). Mém Off Rech sci. tech Outre-mer. 1972; 55:1–289.

[20] Lewis D J. A taxonomic review of the genus *Phlebotomus* (Diptera: Psychodidae). Bull Br Mus nat Hist (Ent). 1982; 45: 121–209.

[21] Talmi-Frank D, Nasereddin A, Schnur LF, Schönian G, Töz SÖ, Jaffe CL, et al (2010) Detection and Identification of Old World *Leishmania* by High Resolution Melt Analysis. PLoS Negl Trop Dis. 2010; 4(1): e581 10.1371/journal.pntd.0000581 20069036 PMC2797090

[22] Schlein Y. Marking of *Phlebotomus papatasi* (Diptera: Psychodidae) by feeding on sprayed, coloured sugar bait: A possible means for behavioural and control studies. Trans R Soc Trop Med Hyg. 1987; 81: 599 3445342 10.1016/0035-9203(87)90421-4

[23] Katholi CR, Unnasch TR. Important experimental parameters for determining infection rates in arthropod vectors using pool screening approaches. Am J Trop Med Hyg. 2006;74(5):779–85. 16687680

[24] Kilpatrick A M, Kramer L D, Campbell S R, Alleyne E O, Dobson A P, Daszak P. West Nile Virus risk assessment and the bridge vector paradigm. Emerg Infect Dis. 2005; 11: 425–429. 15757558 10.3201/eid1103.040364 PMC3298247

[25] Lillie TH, Marquardt WC, Jones RH. The flight range of *Culicoides variipennis* (Diptera: Ceratopogonidae).Can Entomol. 1981; 113 (05): 419–426. 10.4039/Ent113419-5.

[26] White D J, Morris C D. Bionomics of anthropophilic Simuliidae (Diptera) from Adirondack Mountains of New York State, USA: 1. Adult dispersal and longevity. J Med Entomol. 1985; 22(2):190–199. 10.1093/jmedent/22.2.190. 3981559

[27] Lane R P, Fritz G N. The differentiation of leishmaniasis vector *Phlebotomus papatasi* from the suspected vector *P*. *bergeroti* (Diptera: Phlebotominae). Syst Entomol. 1986; 11:439–445.

[28] Wasserberg G, Abramsky Z, Kotler BP, Ostfeld RS, Yarom I, Warburg A. Anthropogenic disturbances occurrence of cutaneous leishmaniais in Israel deserts: patterns and mechanisms. Ecol Appl. 2003; 13:868–881.

[29] Yuval B. Populations of *Phlebotomus papatasi* (Diptera: Psychodidae) and the Risk of *Leishmania major* Transmission in Three Jordan Valley Habitats. J Med Entomol 1991; 28(4): 492–495. 10.1093/jmedent/28.4.492. 1941907

[30] Yuval B, Warburg A, Schlein Y. Leishmaniasis in the Jordan Valley. V. Dispersal characteristics of the sandfly *Phlebotomus papatasi*. Med Vet Entomol 1988; 2:391–395. 2980198 10.1111/j.1365-2915.1988.tb00213.x

[31] Fryauff D J, Modi G B, Mansour N S, Kreutzer R D, Soliman S, Youssef F. Epidemiology of cutaneous leishmaniais at a focus monitored by the multinational force and observes in the Northeastern Sinai Desert of Egypt. Am J Trop Med Hyg. 1993; 49: 598–607. 8250099 10.4269/ajtmh.1993.49.598

[32] Hanafi A H Fryauff D J, Modi G B, Ibrahim M O, Main A J. Bionomics of phlebotomine sandflies at a peacekeeping duty site in the north of Sinai, Egypt. Acta Trop 2007; 101: 106–114. 17270138 10.1016/j.actatropica.2006.12.005

[33] Ali R M, Loutfy N F, Awad O M, Suliman N K. Bionomics of phlebotomine sandfly species in west Alexandria, Egypt. J Entomol and Zool Studies. 2016; 4:349–353.

[34] Reza F M, Mansour N. Entomological studies on *Phlebotomus papatasi* and *P*. *sergenti* (Diptera: Psychodidae) as vectors of cutaneous leishmaniais in Shiraz, Iran. Southeast Asian Trop Med Public Health. 2006; 37(suppl 3): 115–117. 17547064

[35] Morsy T A, Aboul Ela R Gh, Rifaat M M A, Al Dakhil M A. The seasonal and daily activities of *Phlebotomus papatasi* in Riyadh, Saudi Arabia. J Egypt Soc Parasitol. 1995; 25: 699–711. 8586865

[36] Doha A A, Samy A M. Bionomics of phlebotomine sand flies (Diptera: Psychodidae) in the province of Al-Baha, Saudi Arabia. Mem Inst Oswaldo Cruz, Rio de Janeiro. 2010; 105: 850–856. 10.1590/s0074-02762010000700002 21120352

[37] Chelbi I, Derbali M, Al-Ahmadi Z, Zaafouri B, El Fahem A, Zhioua E. Phenology of *Phlebotomus papatasi* (Diptera: Psychodidae) relative to the seasonal prevalence of zoonotic cutaneous leishmaniasis in Central Tunisia. J Med Entomol. 2007; 44: 385–388. 17427714 10.1603/0022-2585(2007)44[385:poppdp]2.0.co;2

[38] Faraj C, Ouahabi S, Adlaoui EB, Elkohli ME, Lakraa L, Rhazi ME et al Insecticide susceptibility status of Phlebotomus (Paraphlebotomus) sergenti and Phlebotomus (Phlebotomus) papatasi in endemic foci of cutaneous leishmaniasis in Morocco. Parasit Vectors. 2012 3 19;5(1):51. 22429776 10.1186/1756-3305-5-51 PMC3359231

[39] Merdan A L, Morsy T A, Shoukry A, Wahba M M. Two successive years studies on Phlebotomus papatasi in North Sinai Governorate, Egypt. J Egypt Soc Parasitol. 1992; 22(1):91–100. 1578184

[40] Wasserberg G, Yarom I, Warburg A. Seasonal abundance patterns of the sandfly Phlebotomus papatasi in climatically distinct foci of cutaneous leishmaniasis in Israeli deserts. Med Vet Entomol. 2003; 17:452–456. 14651661 10.1111/j.1365-2915.2003.00461.x

[41] Berger R, Wasserberg G, Warburg A, Orshan L, Kotler BP. Zoonotic Disease in a Peripheral Population: Persistence and Transmission of *Leishmania major* in a Putative Sink-Source System in the Negev Highlands, Israel. Vector-Borne Zoonot. 2014; 14 (8): 592–600. 10.1089/vbz.2013.1535 PMC4117264 25072990

[42] Janini R, Saliba E, Kamhawi S. Species Composition of Sand Flies and Population Dynamics of *Phlebatomus papatasi* (Diptera: Psychodidae) in the Southern Jordan Valley, an Endemic Focus of Cutaneous Leishmaniasis. J Med Entomol. 1995; 32(6):822–826. 8551505 10.1093/jmedent/32.6.822

[43] Yaghoobi-Ershadi MR, Akhavan AA, Zahraei-Ramazani AR, Jalali-Zand AR, Piazak N. Bionomics of Phlebotomus papatasi (Diptera: Psychodidae) in an endemic focus of zoonotic cutaneous leishmaniasis in central Iran. Journal of Vector Ecology. 2005 6 1;30(1):115 16007964

[44] Faulde MK, Heyl G, Amirih ML. Zoonotic cutaneous Leishmaniasis, Afghanistan. Emerg Infec Diseas. 2006; 12(10): 1623–1624. 10.3201/eid1210.060076 PMC3290938 17176597

[45] Saliba E K, Oumeish OY, Haddadin J, Amr A, Ashford R W. Cutaneous leishmaniasis in Mowaqqar Area, Amman Governorate, Jordan. Ann Trop Med Parasitol.1985; 79:139–146. 3913387 10.1080/00034983.1985.11811900

[46] Orshan L, Szekely D, Khalfa Z, Bitton S. 2010 Distribution and seasonality of phlebotomus sand flies in cutaneous leishmaniasis foci, Judean Desert, Israel. J Med Entomol. 2010; 47: 319–328. 20496578 10.1603/me09096

[47] Faiman R, Kirstein O, Freund M, Guetta H, Warburg A. Exclusion of phlebotomine sand flies from inhabited areas by means of vertical mesh barriers. Trans R Soc Trop Med Hyg. 2011; 105: 512–518. 10.1016/j.trstmh.2011.05.011 21752415

[48] Müller GC, Kravchenko VD, Rybalov L, Beier JC, Schlein Y. Characteristics of resting habitats of adult *Phlebotomus papatasi* in Neot Hakikar, an oasis south of the Dead Sea. J Vector Ecolo. 2011; 36 Suppl. 1: S179–S186. 10.1111/j.1948-7134.2011.00129.x 21366773

[49] Barhoumi W, Qualls WA, Archer RS, Fuller DO, Chelbi I, Cherni S et al Irrigation in the arid regions of Tunisia impacts the abundance and apparent density of sand fly vectors of *Leishmania infantum*. Acta Trop. 2015; 141: 73–78. 10.1016/j.actatropica.2014.10.008. 10.1016/j.actatropica.2014.10.008 25447265 PMC4312219

[50] Wasserberg G, Abramsky Z, Anders G, El-Fari M, Schoenian G, Schnur L et al The ecology of cutaneous leishmaniasis in Nizzana, Israel: infection patterns in the reservoir host, and epidemiological implications. Int J Parasitolo. 2002;32(2):133–43. 10.1016/s0020-7519(01)00326-5 11812490

[51] Killick-Kendrick R, Rioux JA, Baily M, Guy MW, Wilkes TJ, Guy FM et al Ecology of leishmaniasis in the south of France. 20. Dispersal of *Phlebotomus ariasi* Tonnoir 1921 as a factor in the spread of visceral leishmaniasis in the Cévennes. Ann Parsitol Hum Comp. 1984; 59:555–572. 6524821

[52] Alexander B, Young DG. Dispersal of phlebotomine sand flies (Diptera: Psychodidae) in a Colombian focus of leishmania (Viannia) brasiliensis. Mem Inst Oswaldo Cruz. 1992; 87(3): 397–403.‏ 1343649 10.1590/s0074-02761992000300010

[53] Quate LW. Phlebotomus sandflies of the Paloich Area in the Sudan (Diptera, Psychodidae). J Med Entomol. 1964; 1:213–268. 14222867 10.1093/jmedent/1.3.213

[54] Foster WA. 1972 Studies on leishmaniasis in Ethiopia. III. Resting and breeding sites, flight behavior, and seasonal abundance of *Phlebotomus longipes* (Diptera: Psychodidae). Ann Trop Med Parasit. 1972; 66, 313–328. 4634775

[55] Bettini S, Contini C, Atzeni MC, Tocco G. Leishmaniasis in Sardinia. I. Observations on a larval breeding site of *Phlebotomus perniciosus*, *Phlebotomus perfiliewi perfiliewi* and *Sergentomya minuta* (Diptera: Psychodidae) in the canine leishmaniasis focus of Soleminis (Cagliari). Ann Trop Med Parasitol. 1986; 80:307–315. 3800470

[56] Killick-Kendrick R. Breeding places of *Phlebotomus ariasi* in the Cévennes focus of leishmaniasis in the south of France. Parasitologia. 1987; 29:181–191. 3508261

[57] Schlein Y, Yuval B, Jacobson RL. Leishmaniasis in the Jordan Valley: Differential attraction of dispersing and breeding site populations of *Phlebotomus papatasi* (Diptera: Psychodidae) to manure and water. J Med Entomol. 1989; 26:411–413. 2795612 10.1093/jmedent/26.5.411

